# Data of optimization of laccase production by *Marasmiellus palmivorus* LA1 under solid state fermentation using one factor at a time method

**DOI:** 10.1016/j.dib.2018.02.011

**Published:** 2018-02-14

**Authors:** Aiswarya. C, Nayana. P, Padma Nambisan

**Affiliations:** Plant Biotechnology Laboratory, Cochin University of Science and Technology, Cochin-22, India

## Abstract

This article contains data regarding optimization of laccase production under solid state fermentation by the basidiomycetes fungi *Marasmiellus palmivorus* LA1.The data was collected by varying one factor at a time and keeping other factors of the system constant. Influence of environmental factors (substrate type, temperature, inoculums size, substrate weight, substrate length, pH), nutritional factors (organic and inorganic nitrogen sources, carbon sources, surfactants) and elicitors added to media was examined through the laccase activity of the extract. Specific activity at every stage was also measured. The data presents an opportunity to compare the relative influential role of each of the contributing factors on laccase production and directly relates to our research article “Optimization of laccase production from *M. palmivorus* LA1 by Taguchi method of design of experiments” (Chenthamarakshan et al., 2017 [1]).

**Specifications table**

**Table:** 

Subject area	*Biotechnology*
More specific subject area	*Bioprocess technology - Enzyme production.*
Type of data	*Figure*
How data was acquired	*Assaying using UV –visible spectrophotometer (Shimazdu UV-1601)*
Data format	*Analyzed*
Experimental factors	*Mycelium free supernatants were withdrawn periodically as per the factor optimized and analyzed.*
Experimental features	*Laccase activity was determined from the oxidation of ABTS (2, 2’ o-azinobis(3-ethylbenzathiazoline-6-sulfonic acid)) at 25 °C**[2]**and protein content estimation by Folin–Ciocalteu method**[3]*
Data source location	*Not applicable*
Data accessibility	*Data is with this article.*
Related research article	Chenthamarakshan, A., Parambayil, N., Miziriya, N., Soumya, P.S., Lakshmi, M.K., Ramgopal, A., Dileep, A. and Nambisan, P., 2017. Optimization of laccase production from Marasmiellus palmivorus LA1 by Taguchi method of Design of experiments. *BMC biotechnology*, 17(1), p.12. [1].

**Value of the data**

• The data presented in this article supply the optimum condition required for the maximum laccase production under solid state fermentation by *Marasmiellus palmivorus* LA1.
• Time course analysis cumulatively assembled the individual contributing factors for maximum laccase production under the given conditions.
• Use of pineapple leaves, a locally available agroresidue for fermentation makes the process economical and ecofriendly.
• Individual factor identification is an essential preliminary screening step prior to statistical optimization.

## 1 Data

**Fig. 1:**
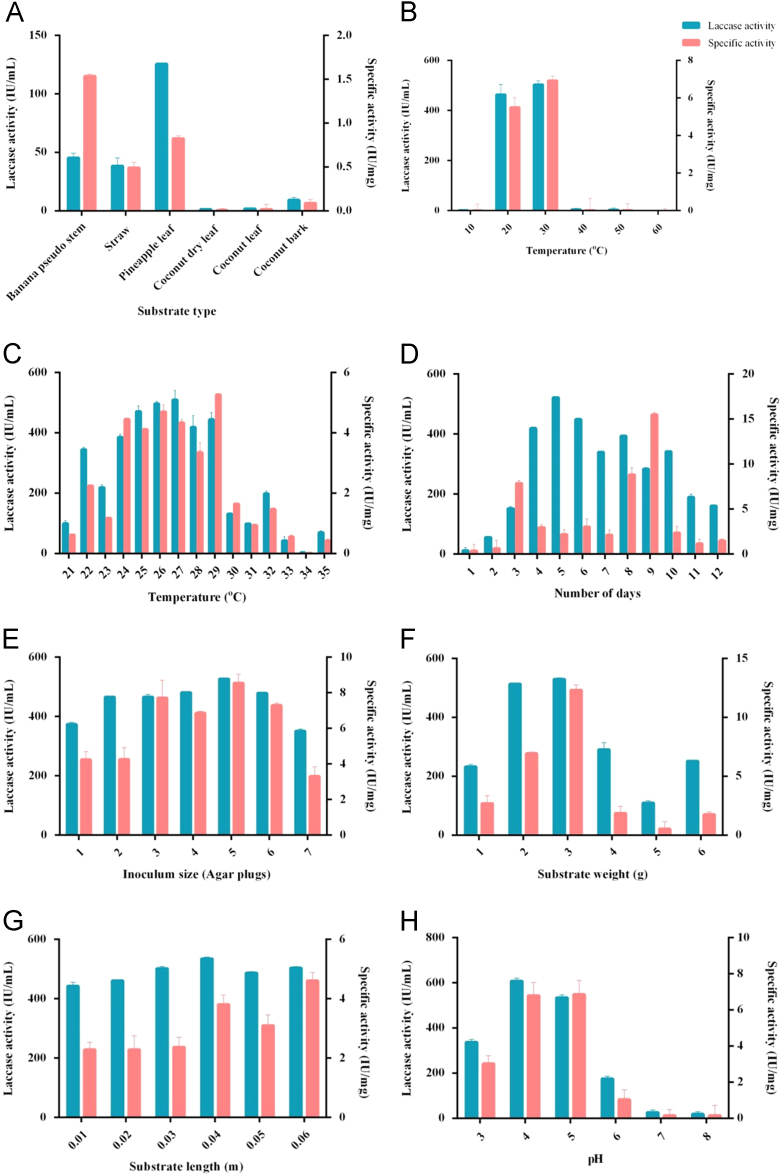
Effect of different physical factors on laccase production by *Marasmiellus palmivorus* LA1. A-Different agroresidues, B-Broad range temperature (°C), C-Selected narrow range temperature (°C), D-Incubation period (days), E-Inoculum size (mycelia agar plugs), F-Pineapple leaf weight (g), G-Pineapple leaf length (m), H- pH.

**Fig. 2:**
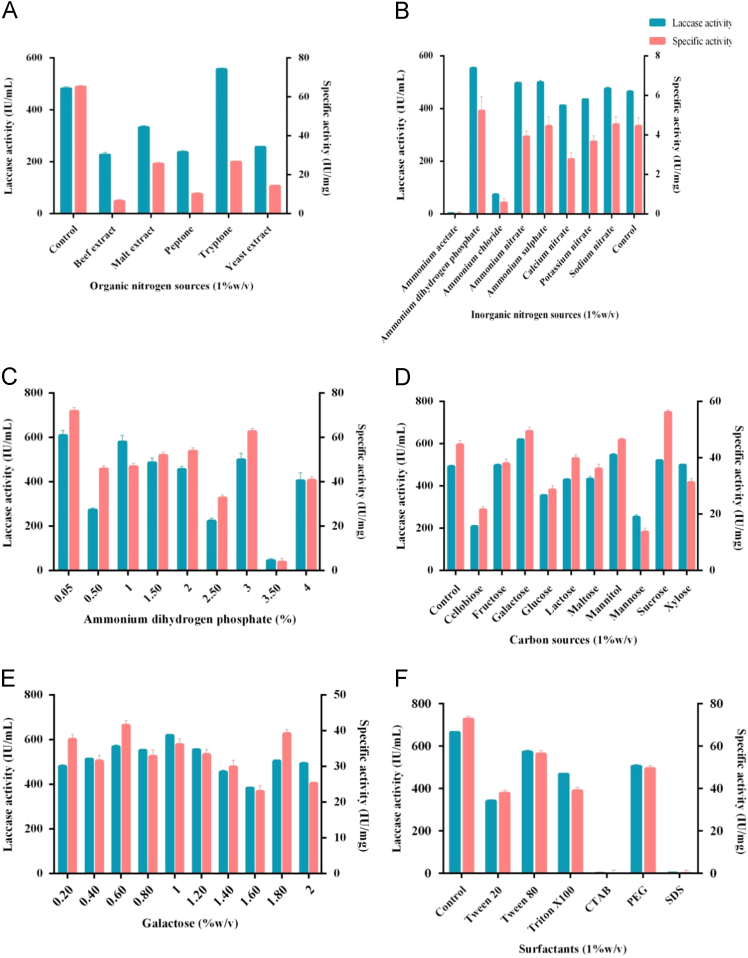
Change in laccase production due to the effect of A-Organic nitrogen sources, B-inorganic nitrogen sources, C-Ammonium dihydrogen phosphate concentration, D-Carbon sources, E- Galactose, F- Surfactants.

**Fig. 3:**
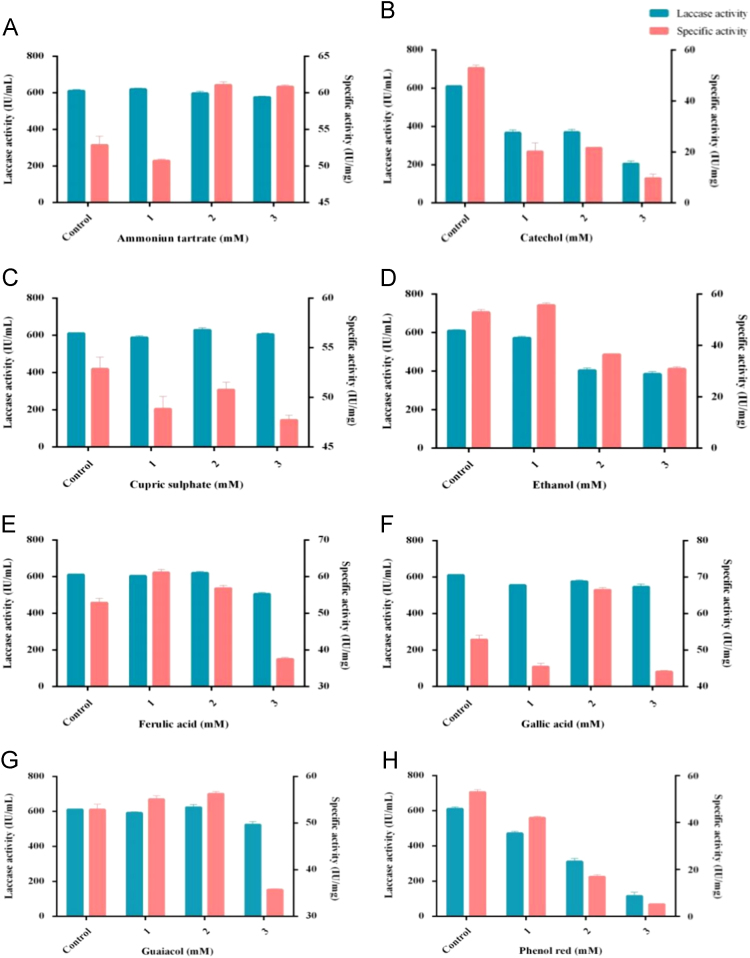

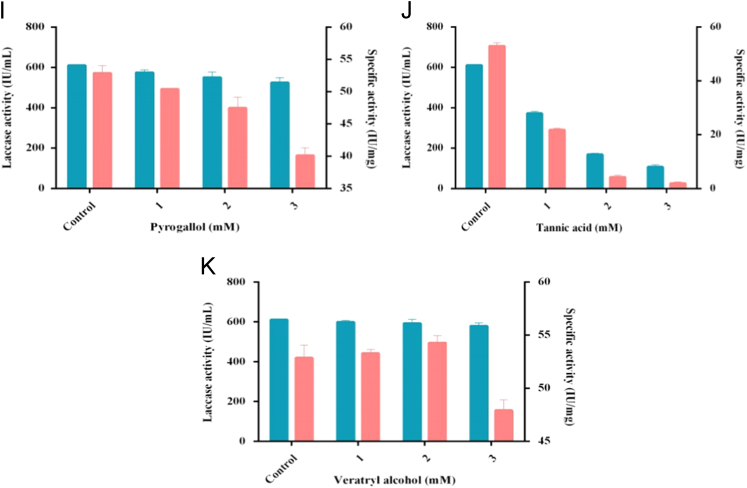
Effect of different inducers at three different concentrations used for laccase production. A- Ammoinum tartrate, B- Cupric sulphate, C- Catechol, D- Ethanol, E-Ferulic acid, F- Gallic acid, G-Guaiacol, H-Phenol red, I-Pyrogallol, J-Tannic acid, K-Veratryl alcohol.

**Fig. 4:**
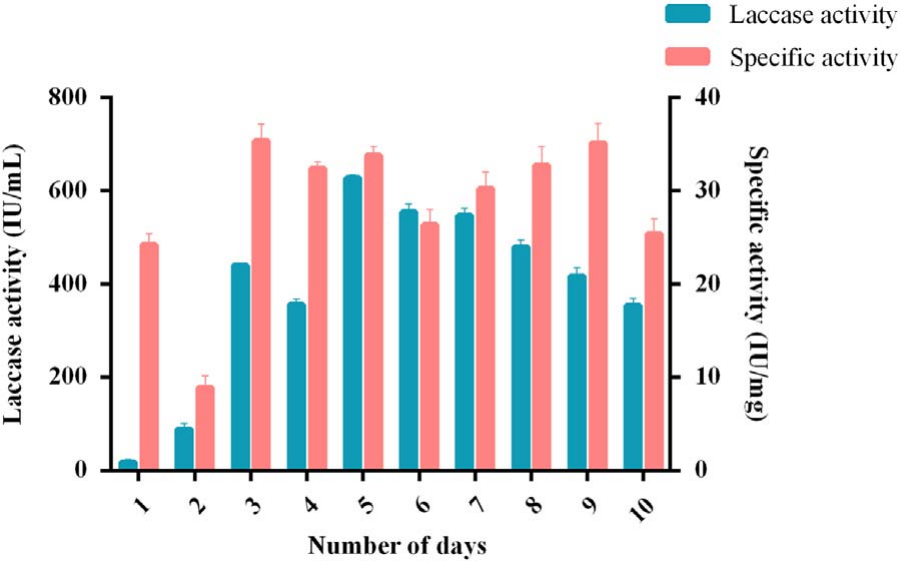
Time course study analysis under optimized conditions for higher laccase production.

A solid state fermentation system with *Marasmiellus palmivorus* LA1 was optimized for maximum laccase production by varying environmental factors (Fig. 1A-H), medium components (Fig. 2A-F), and production elicitors (Fig. 3A-K). All the selected factors were present in time course analysis (Fig. 4), which gives an enzymatic activity of 627.7 IU/mL and specific activity of 33.8 IU mg, using 3 g of 0.04 m long pine apple leaf as substrate with 0.1 M sodium citrate of pH 4 as buffer and 5 agar plugs as inoculum incubated for 5 days at 27 °C with the addition of ammonium dihydrogen phosphate (0.05%), galactose (1%w/v) and cupric sulphate (2 mM).

## 2 Experimental design, materials, and methods

Different conditions affecting laccase production was assessed by growing the *M. palmivorus* LA1 under solid state fermentation with mycelial agar plug inoculum in a 250 mL Erlyenmeyer flask. All the agroresidues were obtained locally and the chemicals from Himedia lab chemicals. During each factor optimization level, all the factors except the particular factor under consideration were made constant. For all the media parameters once the relevant factor was identified, its best suited concentration was also determined. Solid state fermentation with all the factors at optimum condition using *M. palmivorus* LA1 was conducted during time course analysis.

Mycelium free supernatants were withdrawn periodically as per the factor optimized and centrifuged at 9000 g for 10 minutes for extracellular enzyme extraction and then tested for laccase activity with ABTS assay [2]. One unit of enzyme activity was defined as the amount of enzyme required for the conversion of one micromole of substrate per minute under assay conditions. The protein content of mycelium free supernatant was calculated by Folin–Ciocalteu method using bovine serum albumin as standard [3]. The specific activity was calculated by dividing the laccase activity with protein content and was expressed as IU/mg. All the experiments were performed in triplicates. A control was also placed with all the factors present other than the factor under study. The results were graphically represented by Graph Pad Prism, version 6.01.
